# Short-term effect of plant-based Nordic diet versus carbohydrate-restricted diet on glucose levels in gestational diabetes – the eMOM pilot study

**DOI:** 10.1186/s40795-023-00744-7

**Published:** 2023-07-14

**Authors:** Lisa Torsdatter Markussen, Jemina Kivelä, Jaana Lindström, Reza A. Ashrafi, Seppo Heinonen, Saila Koivusalo, Jelena Meinilä

**Affiliations:** 1 Department of Food and Nutrition, University of Helsinki, Helsinki, Finland; 2 Department of IT Management, Helsinki University Hospital, Tukholmankatu 8, Biomedicum 2C, Helsinki, 00029 HUS Finland; 3 Population Health Unit, Finnish Institute for Health and Welfare, Helsinki, Finland; 4 Department of Obstetrics and Gynecology, Helsinki University Hospital and Helsinki University, Helsinki, Finland; 5 Department of Computer Science, Aalto University, Helsinki, Finland; 6 Department of Obstetrics and Gynecology, Turku University Hospital and Turku University, Turku, Finland

**Keywords:** Gestational Diabetes Mellitus, Nordic Diet, Carbohydrate restriction, Medical nutrition therapy, Crossover, CGM, Glycemic control

## Abstract

**Background:**

The optimal nutritional treatment for gestational diabetes (GDM) is still a matter of debate. With increasing rates of GDM and potential negative consequences for the health of mother and child, the best treatment should be established. The Nordic diet with emphasis on plant-based protein show promising health outcomes in other populations but has yet to be investigated in GDM population. The aim of this study, which is part of the “Effect of plant-based Nordic diet versus carbohydrate-restricted diet on glucose levels in gestational diabetes” (eMOM) pilot study was to compare the short-term effects of healthy Nordic diet (HND) and the currently recommended moderate restriction of carbohydrates diet (MCRD) on glucose and lipid metabolism in women with GDM.

**Methods:**

This was a randomized crossover where each of the diet interventions (HND and MCRD) were consumed for 3 days with a 3-day wash-out period in between. In total, 42 pregnant women diagnosed with GDM (< 29 + 0 gestational week) were randomized. Glucose data was collected by continuous glucose monitors (CGM, Freestyle Libre®, Abbott, USA) worn for 14 days, and participants gave blood samples before and after diet interventions. The primary outcome was time spent in glucose target range (TIR, < 7.8 mmol/L). TIR, 3-day mean tissue glucose as well as changes in fasting glucose, homeostatic model of insulin resistance (HOMA-IR) and blood lipids were analyzed with paired samples statistical analyses.

**Results:**

Thirty-six women with complete 14 days CGM data were analyzed. Both diet interventions produced a high degree of TIR (99% SD 1.8), without a difference between the diets (*p* = 0.727). The 3-day mean glucose was significantly lower in HND than in MCRD (*p* = 0,049). Fasting insulin (*p* = 0,034), insulin resistance (*p* = 0,030), total and LDL cholesterol (*p* = 0,023 and 0,008) reduced more in the MCRD diet than the HND. NS differences in any other measure of CGM or blood tests.

**Conclusions:**

HND and MCRD did not differ in terms of their short-term effect on TIR. A larger study with sufficient power is needed to confirm the differences in short-term mean glucose, insulin resistance and lipid metabolism.

**Trial registration:**

Registered in clinicaltrials.gov (21/09/2018, NCT03681054).

**Supplementary Information:**

The online version contains supplementary material available at 10.1186/s40795-023-00744-7.

## Background

Gestational Diabetes Mellitus (GDM) is diabetes that is first diagnosed during pregnancy and is not either type 1 or type 2 diabetes [1, 2]. The rates of GDM worldwide have been increasing mainly due to the increasing prevalence of maternal obesity [3]. Women with GDM have an increased risk for pre-eclampsia, caesarean delivery, and premature delivery [4, 5]. The infants have an increased risk for macrosomia, shoulder dystocia and neonatal hypoglycemia [6]. The International Diabetes Federation estimates that nearly 16% of live births worldwide are affected by hyperglycemia, with GDM represents the majority, over 80% [7]. In 2018 the prevalence of GDM was 21% in Finland [8], which is high compared with other Nordic countries [9].

Currently, the preferred first-line treatment for GDM is dietary intervention for glycemic control [10, 11]. Additionally, daily physical activity (PA) is recommended [10]. Women with GDM generally monitor their glucose levels by fingerstick glucometer (self-monitoring of blood glucose, SMBG), and the treatment goal for GDM is to keep blood glucose within a healthy range to reduce short- and long-term risks of adverse outcomes [10]. However, the optimal nutrition recommendations for women with GDM are still a matter of debate [10], as none of the diets studied has shown superiority in terms of outcomes for the mother or the child [10, 12–16]. One commonly used dietary approach in treating GDM is a restriction of carbohydrates to around 40% of the daily energy intake [15, 17, 18]. However, treatment with carbohydrate-restricted diets lacks high-quality evidence [14, 19], and substituting carbohydrates with energy from fat may have negative consequences for the future health of the fetus [14]. In a recent study, a diet high in complex carbohydrates was more beneficial than a low carbohydrate diet for fasting glucose and a tendency towards lower adiposity of the infants [20].

The Healthy Nordic diet (HND) has a high content of fiber-rich and complex carbohydrate ingredients like whole grains, legumes, berries, fruits and vegetables, polyunsaturated fats from oily fish and a reduced intake of red meat. Eating HND has shown promising results in improved insulin sensitivity for people with type 2 diabetes [21–23]. Moreover, protein intake primarily of animal origin has been associated with lowered insulin sensitivity and increased risk of GDM [24]. Plant-based diets and diets low in animal products are associated with decreased risk of GDM and may also affect insulin sensitivity positively during pregnancy [25]. The effect of protein sources on maternal glycemia is sparsely studied in GDM populations [24]. As such, a dietary regimen beyond carbohydrate restriction is worth exploring.

Therefore, this pilot randomized controlled study, the “Effect of plant-based Nordic diet versus carbohydrate-restricted diet on glucose levels in gestational diabetes” (eMOM)study aimed to compare how the HND with emphasis on plant-based protein compare with the currently recommended moderately carbohydrate-restricted diet (MCRD) on glucose management in women with GDM in a two-week crossover setting and whether a larger trial to test the hypothesis is necessary. The hypothesis of the eMOM pilot study was that a diet high in plant-based protein and Nordic foods without carbohydrate restriction would be more beneficial for glucose management measured by continuous glucose measurement (CGM) compared to a moderately carbohydrate-restricted diet as recommended in the current care guidelines that was valid during this pilot [26].

## Methods

### Study setting and design

Women with singleton pregnancy and a recent diagnosis of GDM (< 29 gestational week, GW) were recruited for the study in the Helsinki Metropolitan area, Finland. The eMOM study had a combination of crossover and parallel study designs, starting after randomization (GW 24–28) with a two-week crossover phase (short-term effects). This was followed by a parallel design randomized controlled trial finishing to delivery (long-term effects). The current paper focuses on the crossover phase and the diets’ short-term effects on glucose and lipid levels.

**Fig. 1:**
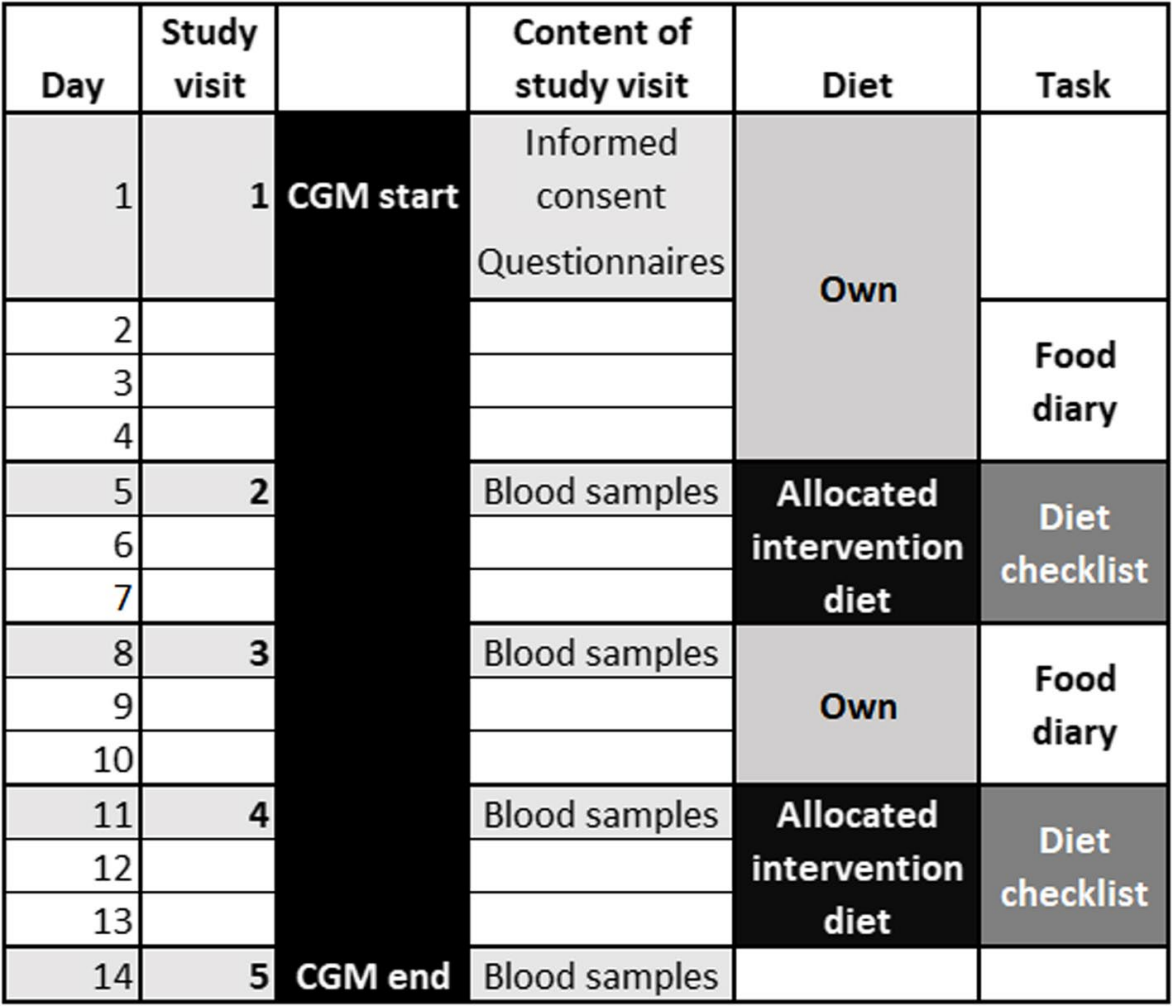
Participant journey crossover flow, schedule, and contents of study visits. All blood samples were drawn in fasted state in the morning. CGM: continuous glucose monitoring

The order of the intervention diets was randomized after eligibility and consenting to participation. The crossover period contained five study visits. The participants consumed their habitual diet during the run-in when entering the study and wash-out periods (between the intervention diets). The crossover schedule and participant flow through the study are presented in Fig. 1.

### Participants and allocation

The women were recruited from the municipal outpatient maternal health clinics of Helsinki, Finland between December 2018 and March 2020, with the last participant completing the study in June 2020. Women were ineligible for participation if they had another type of diabetes than GDM, used medication that influenced glucose (e.g. oral corticosteroids or metformin/insulin) or cholesterol metabolism, had dietary restrictions that interfered with the intervention, they or the child’s father had other than European ethnicity, or had issues interfering with participation (e.g., inadequate Finnish language skills or substance abuse). The number of participants (aim *n* = 60) was decided based on the available resources and to provide large enough number of participants at the end of the entire eMOM study, including the parallel study design that followed the crossover phase. Recruitment to the study took more time than planned and was stopped due to COVID-19 restrictions and reaching the end of the funding period.

To randomly assign participants to treatment arms, randomization with blocks of four was completed using a web page (URL: https://www.sealedenvelope.com), separately for nulliparous and multiparous women. The diet allocation was concealed in opaque sealed envelopes with continuous numbering. The envelopes were prepared by a researcher who did not meet the participants upon enrolment and allocation. The study personnel and participants were not blinded for diet allocation during the study because it was not possible considering the setting of the study. The data analyst was blinded for diet allocation during analysis.

If a participant needed pharmacological treatment (metformin or insulin) to maintain normal blood glucose, they were advised to contact their maternity health clinic for treatment and their participation in the study was discontinued.

### Interventions

All foods and meals for the two 3-day crossover dietary periods were provided for the participants and their spouses/partners (to enhance adherence). Nutritionists planned the menus in collaboration with restaurant chef students and their teachers. The provided meals for both diets included 2200 kcal per day and participants got breakfast, two ready-made hot main meals, afternoon snack and evening snack daily. The goal macronutrient composition of both intervention diets is provided in Table 1. The full list of provided foods and meals and their total macronutrient contents are available in "Supplemental Table 1”.

**Table 1:** Provided macronutrient characteristics of the MCRD and HND diets

	Moderately carbohydrate restricted	Healthy Nordic Diet
Energy, kcal	2200	2200
Protein, E%	20	20
Carbohydrates, E%	40	55
Sucrose, E%	< 10	< 10
Fat, E%	40	25
Saturated fatty acids, E%	< 10	< 10
Polyunsaturated fatty acids, E%	5–10	5–10
Dietary fiber, g	32	36

*HND* Healthy Nordic Diet, *MCRD* Moderately carbohydrate-restricted, *E%* Percentage of the total energy intake

The Finnish Nutrition recommendations and mild carbohydrate restriction, which is currently (and was at the time of the study) recommended for GDM treatment in Finland [17], were the basis for the MCRD. The meals included mainly high fiber carbohydrates, for example brown rice, whole grain toast, oatmeal porridge and brown pasta. Meals contained around 0.5 kg of fruits and vegetables daily. Poultry and low-fat dairy products were the main protein sources in addition to two red-meat meals in a 3-day period and protein from grains. Vegetable oils and vegetable oil spreads were the main fat sources. In addition, meals included fat from meat products and a few portions of nuts were provided.

The HND was characterized by Nordic ingredients, plant-based protein sources, and complex carbohydrates without restriction. The main sources of carbohydrates were of Nordic whole grains (oats, barley and rye) as bread, porridge, pasta and cooked grains. Meals contained at least 1 kg of berries, fruits and vegetables daily with emphasis on Nordic species (e.g., bilberries, lingonberries, apples, onion, kale, and carrots). Legumes and processed plant-protein products (mainly fava beans), fermented low-fat dairy and fish (two fish meals for 3 days) were the main protein sources. Rapeseed oil and vegetable oil spread were the main sources of fat. In addition, small portions of nuts were included in some snack meals.

The participants marked their adherence with the diets in checklists. Checklist markings included eating time, if the provided meals were eaten fully, partially or not at all. Participants also marked if there were any changes to the meal plan, or if any extra items were consumed.

### Outcomes

The primary outcome measure of this study was the percentage of time spent within the target blood glucose range (TIR, < 7.8 mmol/L) [17] measured by CGM (Freestyle Libre®, Abbott, USA) during two 3-day intervention diets. Secondary outcome measures were 3-day mean and standard deviation of glucose and change in blood lipid markers and homeostatic model assessment of insulin resistance (HOMA-IR) after the two 3-day intervention diets.

### Data collection

#### CGM sensor

The CGM sensor (Freestyle Libre®, Abbott, USA) was attached to the participant’s upper arm at the first study visit. The diet intervention period started on the third day after CGM attachment to allow the sensor to adjust. The CGM sensor automatically measures the tissue glucose concentration every minute, and data are stored at a frequency of 15 min. The sensor is coupled with a hand-held reader, which shows the current tissue glucose concentration, a glucose curve of the last 8 h and glucose measurement history. The sensor needs to be scanned with the reader at a minimum every 8 h to collect the glucose data. The participants were instructed to scan the reader at least once during the daytime, before going to bed, and first thing in the morning.

#### Blood samples

Laboratory tests performed in conjunction with study visits included 10–12 h fasting measurements of glucose metabolism (plasma glucose and insulin) and blood lipids (total cholesterol; high-density lipoprotein, HDL; and low-density lipoprotein, LDL; triglycerides, TG). Samples were drawn at the study center in the morning the first day of each diet, and the morning following each diet end, in total four times. Venous blood samples were taken in a seated position with a light stasis and centrifuged at the survey site. Glucose samples were collected in 5 ml fluoride citrate vacuum tubes and analyzed by photometric hexokinase method. Insulin samples were collected in 5 ml serum gel tubes, centrifuged after collection, and analyzed with the immunochemiluminometric method. All blood samples for lipid markers (total cholesterol, HDL, LDL and TG) were collected in 5 ml li-heparin tubes, centrifuged, and analyzed with a photometric enzymatic method. All samples were analyzed by the Helsinki University Hospital laboratory (HUSLAB).

#### Questionnaires and baseline demographic data

All participants filled out a baseline questionnaire about demographic characteristics (age, educational level, social and work status, income level and profession), the current health status, habits (smoking, alcohol use; AUDIT [27]) and health history, use of medication and the history of diabetes and other illnesses in the close family. PA and activity level were self-rated using a standardized questionnaire commonly used in Finnish nationwide studies [28]. Height and weight at baseline were measured by the study nurse and pre-pregnancy weight was collected from the maternity clinic patient card, in which standard pregnancy-related information is collected from and recorded for the pregnant women.

### Data

#### Analysis and data preparation

The CGM glucose data that covered the days when the participant was following the diet protocols were extracted and preprocessed with Python 3 programming language [29]. The first measurement day was defined as started at 00:00 and the last measurement day ended at 23:59. TIR was calculated by counting the number of measurements that was ≤ 7.8 mmol/l and dividing it by the total number of measurements for each individual, creating a value representing the percentage of the total time. The individual participants 3-day mean, standard deviation of the 3-day glucose, the minimum and the maximum glucose values were calculated and added to a data frame (new variables) for further analysis of the various glucose measurements during the diets. Each individuals’ 3-day CGM data length (i.e. number of observations) was inspected. If a participant had significantly lower than expected amount of glucose observations (> 20% data loss), their glucose curve was inspected. For two participants, there was significant loss of CGM data for longer time frames in one or both diet periods and these participants were excluded from analyses due to poor data quality.

All laboratory measures were entered to the database as double entries. All numerical data was checked for unusual values (i.e. outliers), and in case of unusual or missing values the original data was checked and corrected. A few cases of missing data were confirmed: missing blood sample data (one lipid panel and one fasting insulin), one participants parity and OGTT data was confirmed as not available and as such, not included in the respective analyses (highlighted in the results tables below). If participants had one or more family members (parents or grandparents) with any form of diabetes, this was coded with yes/no. Body mass index (BMI) was calculated by [weight in kilograms/(height in meters)^2^]. HOMA-IR value was calculated from fasting insulin and glucose using the following formula [fasting insulin (mU/L) × fasting glucose (mmol/L)/22.5]. Homeostatic model for beta cell function (HOMA β) was calculated using the following formula [20 × fasting insulin (mU/L)/fasting glucose (mmol/L) - 3.5] [30]. Delta variables (Δ) to analyze postintervention changes were calculated for all blood test results and HOMA indices by subtracting the post-test value from the pre-test value and related to both periods of the crossover.

### Statistical analyses

**Fig. 2:**
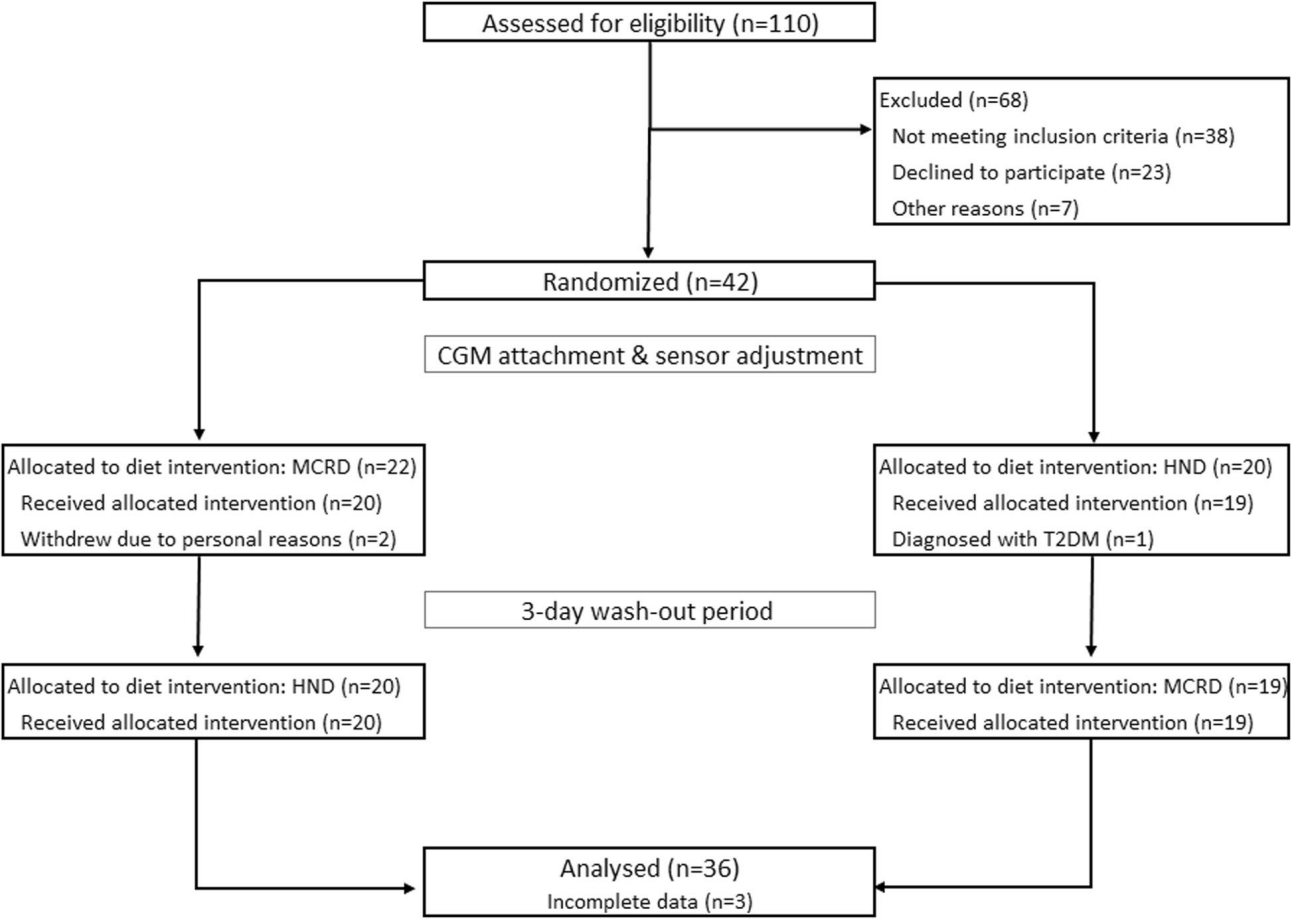
CONSORT flow chart. CGM: continuous glucose monitoring, HND: Healthy Nordic diet, MCRD: moderately carbohydrate restricted, T2DM: type 2 diabetes mellitus

As this was a pilot study, no power calculation was made before the study. A per-protocol analysis approach was used, i.e., participants with incomplete CGM or checklist data were removed from the analysis (three participants, see CONSORT flow chart, Fig. 2). All data were analyzed for normal distributions by the Shapiro-Wilks test and by inspecting the distributions of the data with density and Q-Q plots. Paired t-test and paired Wilcoxon test was utilized to compare the intervention diets (each participant as their own control). A p value lower than 0.05 was considered statistically significant. All statistical analyses were performed with R programming language (version 4.0.5, The R Foundation for Statistical Computing, Vienna, Austria. URL https://www.R-project.org/) in RStudio (version 1.4.1106, “Tiger Daylily”, RStudio: Integrated Development for R. RStudio, PBC, Boston, MA, USA. URL: http://www.rstudio.com/). A sensitivity analysis was performed by removing one participant that was identified as having a larger difference in TIR (89% TIR in MCR, 94.8% TIR in HND, or 5.8% difference) between the diets compared to any other participant. Then, all analyses were repeated.

#### Adherence calculations and analysis

For full adherence to the diets, drinking water, coffee and tea was allowed, and minor switches between similar foods were approved e.g., pear instead of apple, cucumber instead of tomato. For adherence analysis, an adherence score was calculated. Each component of every meal in the diet checklist was given 1 point if consumed fully and 0 if omitted (apart from the exceptions mentioned above). If consumed partially, it was given 0.5 points. The points were summed separately for each day, and a percentage of adherence for each day was calculated based on the total points and the number of food items in all the meals. Spices or comparable items did not count towards the adherence points. If the participant consumed food items other than in the protocol or if meal items had been exchanged with non-protocol adhering items, these were given minus points (minus 1 to minus 0.25 points depending on the size of the serving) and redacted from the day’s total points. Given that the participants were of different body size, adding another serving of protocol-approved food items were not given minus points.

## Results

Out of 42 randomized participants, 36 women had complete CGM and diet checklist data for the crossover period for analysis (detailed view of exclusions in Fig. 2). The women were on average 33.0 (SD 4.3) years old, had a pre-pregnancy weight of 73.7 (SD 14.1) kg and pre-pregnancy BMI of 26.4 (SD 4.4) kg/m^2^. Nineteen (54.3%) of the participants had been diagnosed with GDM prior to GW 24 (Table 2). The majority of the participants were categorized as regularly physically active with light activities (e.g., walks, household and garden chores, several hours weekly, *n* = 21, 58.3%).

**Table 2:** Baseline characteristics of the participants, *n* = 36

	Mean	SD
Age, years	32.7	4.5
Pre-pregnancy weight, kg	74.3	13.0
Height, cm	166.4	5.3
BMI^a^, kg/m^2^	26.7	4.2
GW at randomization, weeks	26.6	1.5
OGTT result^b^, mmol/L^c^		
*Fasting*	5.3	0.4
*1 h*	8.4	2.2
*2 h*	7.2	1.7
Fasting glucose^b^, mmol/l	5.2	0.4
Fasting insulin, mU/L	10.9	4.9
HOMA IR	2.6	1.3
HOMA β	135.6	61.5
Blood lipids		
*Total cholesterol*	*6.0*	*1.0*
*LDL cholesterol*	*3.5*	*0.9*
*HDL cholesterol*	*1.93*	*0.42*
*Triglycerides*	1.91	0.58
	N	%
Early vs. late diagnosis, n (%)^c^		
*Early (*< *24 GW)*	*20*	*57.1*
Parity, n (%)^c^		
*0*	20	57.1
*1*	13	37.1
*2*	2	5.7
Income, household		
< *50 000€/year*	9	25
> *50 000/year*	27	75
Educational level		
*Vocational, high school and polytechnic degree*	13	36.1
*University degree*	23	63.9
Work status		
*Working (full/part time, freelance)*	29	80.6
*Not working (sick/maternity leave, Unemployed, stay home mom)*	6	16.7
*Student*	1	2.8
Family status		
*Single*	2	5.6
*Partner, no children*	19	52.8
*Partner and child(ren)*	14	38.9
*Single with child(ren)*	1	2.8
Smoking		
*No*	29	80.6
*Yes, occasionally*^d^	5	13.9
*Yes, before pregnancy but not during*	2	5.6
Family history of diabetes		
*Parent(s) with diabetes, any type*	10	27.8
*One or more grandparents with diabetes, any type*	17	47.2

*BMI* Body mass index, *GW* Gestational week, *OGTT* Oral glucose tolerance test

^a^Based on pre-pregnancy weight

^b^OGTT performed prior to entering the study, baseline fasting glucose sample drawn at start of study

^c^*N* = 35

^d^Number of cigarettes/tobacco product used was very low, or smoking ended during first half of pregnancy

### TIR and results of CGM

**Fig. 3:**
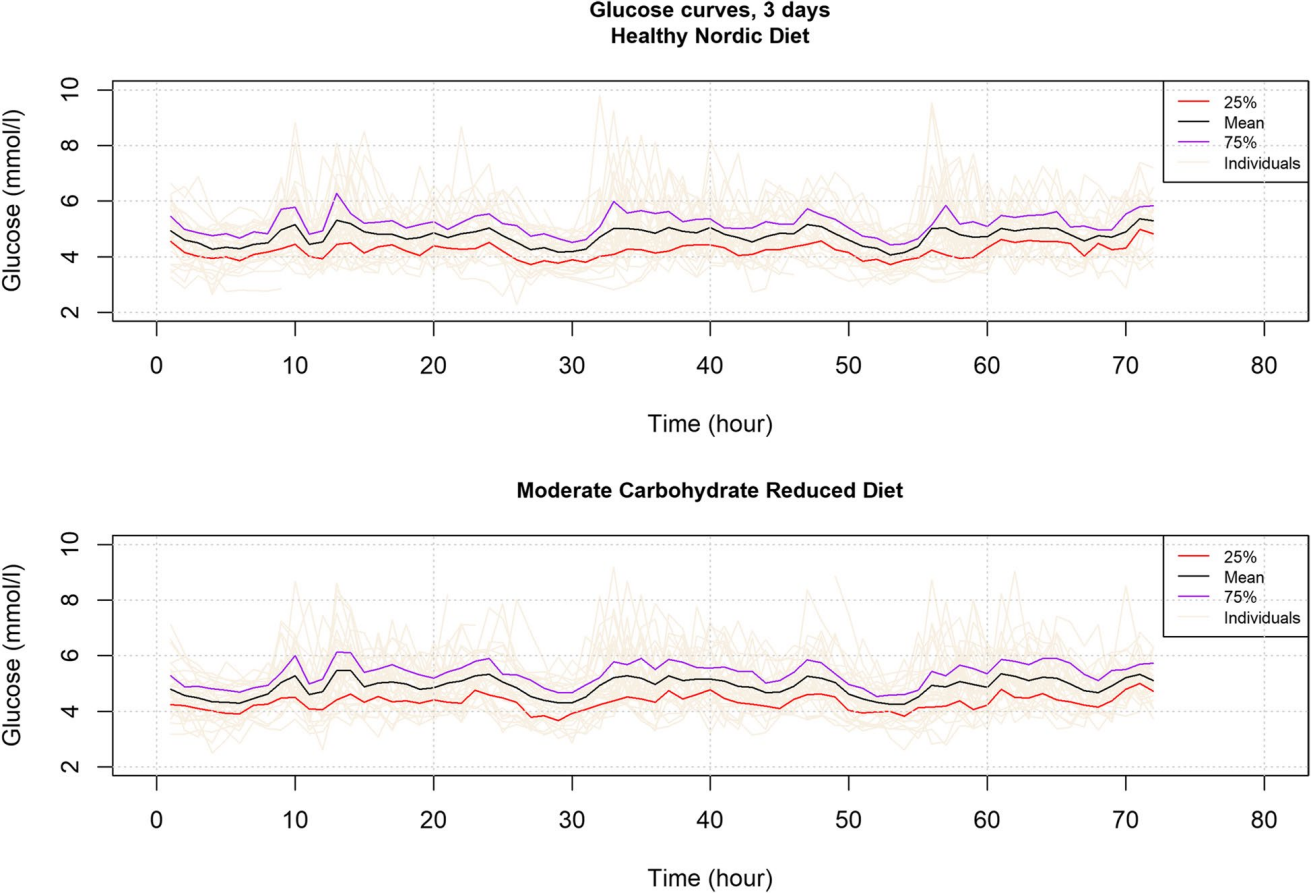
Continuous glucose monitoring curves during both 3-day intervention diets. The mean glucose of all participants (black line), and the 25% (red) and 75% (purple) quartiles are shown plotted in 60 min intervals. Additionally, the individual glucose curves are plotted in beige lines. The CGM time periods started at 00:00 and ended 72 h later at 23:59. CGM: continuous glucose monitoring

The HND diet did not differ from the MCRD in glucose TIR. During both diets, tissue glucose levels were within the range of < 7.8 mmol/L for 99% (SD 1.8) of the time. There was a small, but significantly lower 3-day mean glucose in the HND compared to the MCRD (4.8 SD 0.5 vs. 4.9 SD 0.5 mmol/L, *p* = 0.049). The tissue glucose variability was similar in both diets (Fig. 3); the 3-day standard deviations of glucose in both 3-day periods were similar (Table 3).

**Table 3:** Time in glucose target range (< 7.8 mmol/l) and other glucose data derived through continuous glucose monitoring during each intervention diet and the *p*-value of the difference between the diets

	**MCRD**		**HND**		*P*
	Mean	SD	Mean	SD	
Time in range, %	98.7	2.1	98.9	1.5	0.727
Mean 3-day glucose, mmol/L	4.9	0.5	4.8	0.5	0.049
SD of 3-day glucose, mmol/L	0.9	0.2	0.8	0.2	0.405
Minimum glucose, mmol/L	3.3	0.63	3.2	0.5	0.772
Maximum measured glucose, mmol/L	8.2	1.19	8.2	1.2	0.695

*HND* Healthy Nordic diet, *MCRD* Moderately carbohydrate restricted, *SD* Standard deviation

#### Fasting glucose, insulin resistance and blood lipids

There were no differences between MCRD and HND in fasting blood glucose. Fasting insulin and HOMA-IR had decreased significantly more after the MCRD diet than in the HND diet, but no difference in the change of HOMA β (Table 4) was observed. A significantly greater decrease in both total and LDL cholesterol was found after the MCRD diet, as compared to no change after HND. All other blood lipids remained unchanged after both diet interventions (see Table 4 for full details).

**Table 4:** Blood sample results and changes (**Δ**) in blood levels from before to after intervention diet periods and *p*-value for the difference between the change after each diet

	**MCRD**			**HND**			***P*-value**
	**Pre**	**Post**	**Δ**	**Pre**	**Post**	**Δ**	
	Mean (SD)	Mean (SD)	Mean (95% CI)	Mean (SD)	Mean (SD)	Mean (95% CI)	
Fasting plasma glucose, mmol/L	5.2 (0.4)	5.1 (0.3)	-0.1 (-0.2 to 0.0)	5.1 (0.4)	5.1 (0.3)	0.0 (-0.1 to 0.1)	0.184
Fasting serum insulin, mU/L^a^	11.5 (5.5)	10.1 (4.4)	-1.3 (-2.4 to -0.3)	10.8 (5.1)	10.8 (5.0)	-0.0 (-0.8 to 0.8)	0.034
HOMA-IR^a^	2.67 (1.43)	2.3 (1.05)	-0.37 (-0.68 to -0.06)	2.49 (1.29)	2.46 (1.17)	0.03 (-0.24 to 0.18)	0.030
HOMA β^a^	142 (65.2)	132 (58.5)	-9.97 (-21.54 to 1.95)	138 (62.9)	140 (75)	2.21 (-14.28 to 18.70)	0.334
Total cholesterol, mmol/L^a^	5.9 (1.0)	5.7 (1.0)	-0.2 (-0.4 to -0.1)	5.8 (0.9)	5.8 (1.0)	-0.0 (-0.2 to 0.1)	0.023
LDL cholesterol, mmol/L^a^	3.5 (0.9)	3.3 (0.9)	-0.2 (-0.3 to -0.1)	3.4 (0.9)	3.4 (0.9)	-0.0 (-0.2 to 0.1)	0.008
HDL cholesterol, mmol/L^a^	1.90 (0.44)	1.81 (0.42)	-0.09 (-0.13 to -0.06)	1.90 (0.44)	1.87 (0.44)	-0.03 (-0.08 to 0.01)	0.080
Triglyceride, mmol/L^a^	1.95 (0.67)	1.89 (0.64)	-0.05 (-0.17 to 0.06)	1.89 (0.6)	1.87 (0.65)	-0.01 (-0.11 to 0.09)	0.590

Pre and post represent fasted blood samples sampled on the first day (pre) and the day after (post) each of the 3-day diet periods

*HDL* High density lipoprotein, *HND* Healthy Nordic diet, *HOMA β* Homeostatic model assessment of β-cell function, *HOMA-IR* Homeostatic model assessment of insulin resistance, *LDL* Low density lipoprotein, *MCRD* Moderately carbohydrate restricted, *Δ* Delta values based on post-diet blood sample minus pre-diet blood sample

^a^Blood sample result not available from all participants, *n* = 35

### Sensitivity analysis

In sensitivity analysis excluding a participant with diverging glucose values, the primary finding became further attenuated; there was no significant difference for TIR between the diets (*p* = 1.000). However, with removal of this participant, the 3-day mean glucose became non-significant and lowered the 3-day mean slightly in both diets (MCRD mean = 4.9 SD 0.5, HND mean = 4.7 SD 0.5, *p* = 0.062). None of the other CGM-derived glucose variables or lipid values changed in significance compared to the original findings.

There was no difference in compliance between the diets (0.73 SD 0.34 for MCRD and 0.77 SD 0.13 for HND, *p* = 0.208, 95% CI = -0.08 to 0.02). No harms or unintended effects during the intervention were reported.

## Discussion

This short-term crossover study during the early third trimester suggests that MCRD and HND do not have different effects on glucose TIR. During both diets, the glucose levels remained in the target range for 98% of the time. A small, significantly lower 3-day mean glucose was seen during HND. Conversely, some of the cholesterol values, fasting insulin and HOMA-IR decreased more after the MCRD diet compared to the HND.

To our knowledge, there are no previous studies investigating the Healthy Nordic Diet and glycemic control in GDM, although one is ongoing [31]. According to our study, the MCRD and plant-based HND were both producing satisfactory glucose levels in short term. Both diets had healthy fatty acid profiles, contained a low amount of refined carbohydrates and a high amount of dietary fiber. These diet components have been shown to reduce the chances of hyperglycemia and to be positively associated with glycemic control in women with GDM [19, 32, 33] and other populations [21]. This might explain the lack of difference in TIR between the diets. Although the accurate glycemic index (GI) profile of the diets has not been calculated, the majority of the foods in both diets could be categorized as low to moderate. Diets with lower GI have been beneficial for maternal glycemia in GDM [32, 33]. The short duration of the intervention and the similarity of the intervention diets in terms of healthiness could together be the reason for the lack of differences in TIR.

The 3-day mean glucose was the only CGM glucose variable with statistical significance which could suggest a difference between the diets, favoring the HND condition. This could reflect the favorable effect of legumes on glucose values found in patients with type 2 diabetes [34]. However, the difference in 3-day mean glucose between the diets became non-significant in the sensitivity analysis, suggesting that the significant finding was weak. Overall, the differences in glucose levels both before and after the sensitivity analysis were small. Additionally, during both diets, the 3-day mean glucose levels were below 5 mmol/L. This is likely a reflection of the high dietary quality of both of the diets. Increased consumption of carbohydrates in the HND did not increase any of the glucose variables and kept the mean glucose lower than the diet with less carbohydrates. The HND had the additional benefit of including carbohydrates from legumes, which have been beneficial for glycemic control in type 2 diabetic populations [35].

Both the change in fasting insulin and (as a result) the HOMA for insulin resistance decreased more in the MCRD diet while it remained unchanged for the HND. The increased intake of dietary fat in the MCRD diet did not correspond to a disadvantageous blood lipid profile short term. These results are in line with studies in patients with type 2 diabetes; low-carbohydrate diet has been beneficial for glycemic control and blood lipids in type 2 diabetes [36–38]. Considering the short duration of the intervention of this study, the long-term impacts of the diets remain to be seen.

### Strengths and limitations

We used a crossover design, where each participant served as her own control which minimizes effect of various confounders in the effect of diet on glucose control (e.g. individual sleeping pattern or micronutrient status at baseline) [39]. We provided all the foods for the intervention diet periods, which eased the compliance to the diet and ensured similarity of diets between the participants. Another strength was recording the true compliance of the participants to the diets. Continuous glucose monitoring allowed more comprehensive understanding of glucose management compared to infrequent blood glucose measures available in standard care. This was also the first study to evaluate the effects of plant-based healthy Nordic diet on GDM management.

However, this was a feasibility pilot study without a power calculation and thus did not provide firm conclusions on the differences in results between the diets. The short duration of the study allowed conclusions only on short-term effects. As it would be unethical to include a no-treatment control group, it is not possible to determine the effect of a diet compared to an untreated GDM pregnancy [40, 41]. The use of the unmasked CGM in this study likely also had also an effect on the results. The women were able to monitor their tissue glucose at all times and were able to adjust accordingly, for example, change their meal timing or go for a walk to aid the immediate glucose control. However, this was similar during both diet periods and, thus, is not expected to have distinct effects between the diets.

It is assumed that the underlying mechanism of GDM in women with normal weight is more often insulin deficiency whereas the mechanism of GDM in women with obesity is more often high insulin resistance [42]. In our study population, the proportion of women with normal weight and overweight was similar (41% and 44%, respectively), and a smaller proportion of women were obese. The underlying mechanisms of GDM development have likely been heterogenic in our study population which might have influenced the results concerning glucose control (3 day mean tissue glucose), insulin resistance (fasting insulin and HOMA-IR) and the cholesterol metabolism markers. Therefore, the current results may not be replicated in populations with high BMI.

## Conclusions

In this short-term feasibility pilot with crossover design, we could not confirm our hypothesis as the HND and the currently recommended MCRD did not differ in their short-term effect on TIR in women with GDM. Although a small number of women were included in this pilot, the results suggest that HND may be as good as conventional carbohydrate restriction in maximizing TIR for the treatment of GDM in short term. However, a study with sufficient power is needed to confirm the differences in short-term mean glucose, insulin resistance and lipid metabolism. Whether there are long-term differences in the effects on the glucose management of the mother or the body composition of the offspring, remains to be studied.

## Supplementary Information

**Additional file 1: Supplemental Table 1a.** Moderately carbohydrate restricted diet meals during crossover phase. **Supplemental Table 1b.** The goal and the actual values of macronutrients and salt in crossover meals of Moderately carbohydrate restricted diet, per day. **Supplemental Table 2a.** Healthy Nordic Diet meals during crossover phase. **Supplemental Table 2b.** The goal and the actual values of macronutrients and salt in crossover meals of Healthy Nordic diet, per day.

## Data Availability

The datasets generated and analyzed during the current study are not publicly available due to data protection principles. However, the data are available from the corresponding author upon reasonable request.

## Abbreviations

GDM: Gestational Diabetes Mellitus
CGM: Continuous glucose monitoring
HND: Healthy Nordic Diet
MCRD: Moderately Carbohydrate Restricted Diet
TIR: Time In Range
GW: Gestational week
LDL: Low density lipoprotein
HDL: High density lipoprotein
GI: Glycemic index
PA: Physical activity
SMBG: Self-monitoring of blood glucose
TG: Triglyceride
BMI: Body Mass Index
HOMA IR: Homeostatic model for insulin resistance
HOMA β: Homeostatic model for beta cell function
