# Supplementary material for: Short-term effect of plant-based Nordic diet versus carbohydrate-restricted diet on glucose levels in gestational diabetes – the eMOM pilot study
Source: BMC Nutr. 2023 Jul 14;9:87. doi: 10.1186/s40795-023-00744-7 (PMC10349459; doi:10.1186/s40795-023-00744-7)
Supplement: Supplementary file 1 — Additional file 1: Supplemental Table 1a. Moderately carbohydrate restricted diet meals during crossover phase. Supplemental Table 1b. The goal and the actual values of macronutrients and salt in crossover meals of Moderately carbohydrate restricted diet, per day. Supplemental Table 2a. Healthy Nordic Diet meals during crossover phase. Supplemental Table 2b. The goal and the actual values of macronutrients and salt in crossover meals of Healthy Nordic diet, per day. [file 40795_2023_744_MOESM1_ESM.docx]

**Supplemental tables**

Supplemental table 1a and 1b describe the foods and their amounts in the Moderately carbohydrate restricted diet (MCRD) and supplemental tables 2a and 2b describe the foods and their amounts in the Healthy Nordic diet (HND).

Tables a) provide foods for each diet, and b) each intervention day macronutrient breakdown and diet goal, and price of diet.

Supplemental Table 1a. Moderately carbohydrate restricted diet meals during crossover phase

|  | Day 1 | Day 2 | Day 3 |
| --- | --- | --- | --- |
| Breakfast | Overnight Oats, 200 g  Apple, 120 g | Barley milk porridge, berries, 330 g  Rye bread, margarine, tomato, 43 g | Oatmeal porridge, 300 g  Apple, 120 g |
| Lunch | Rice-chicken wok, 420 g  Cottage cheese, 40 g | Chicken soup, 450 g  Cottage cheese, 80 g  Rye bread, margarine, tomato, 43 g | Tomato minced meat sauce with whole grain pasta, 440 g |
| Snack | Viili (Finnish fermented milk product), apple, bilberries and nuts, 310 g  Rye bread, margarine, tomato, 73 g | Overnight oats with berries, 240 g  Banana, 140 g | Berry-banana-smoothie, 320 g  Whole grain rye bun, pesto, tomato, 70 g |
| Dinner | Chicken filet with filling, 200 g  Vegetable bulgur, 180 g  Tomato sauce, 94 g  Green beans, 50 g | Minced meat patty with avocado filling, 180 g  Oven baked sweet potato, 165 g  Sour cream sauce, 50 g  Green beans, 50 g | Chicken with lime, 210 g  Barley with Indian spices, 210 g  Cilantro yogurt sauce, 50 g |
| Evening snack | Cashew nuts, 35 g  Whole grain rye bun, pesto, tomato, 120 g | Cashew nuts, 35 g  Whole grain rye bun, pesto, tomato, 120 g | Rye bread, pesto, tomato, 150 g  Apple, 120 g |

Supplemental Table 1b. The goal and the actual values of macronutrients and salt in crossover meals of Moderately carbohydrate restricted diet per day

|  | Goal | Day 1 | Day2 | Day 3 |
| --- | --- | --- | --- | --- |
| Price, € | 7€ | 8.34€ | 7.68€ | 5.93€ |
| Energy, kcal | 2200 | 2209 | 2193 | 2217 |
| Carbohydrates, E% | 40 | 40 | 41.7 | 40.8 |
| Protein, E% | 20 | 20.6 | 19.9 | 19.7 |
| Fat, E% | 40 | 39 | 38.7 | 38.6 |
| Salt, grams | ~5 | 4.5 | 5.8 | 5.2 |
| Fiber, grams | > 30 | 33.4 | 36.8 | 38.1 |

Supplemental table 2a and 2b: Healthy Nordic Diet (HND).

Supplemental Table 2a. Healthy Nordic Diet meals during crossover phase

|  | Day 1 | Day2 | Day 3 |
| --- | --- | --- | --- |
| Breakfast | Overnight oats, 200 g  Apple, 120 g | Oatmeal porridge and bilberries, 360 g  Oat bread, pesto, tomato, 78 g | Barley porridge cooked with milk, berry mix, 290 g  Apple, 120g |
| Lunch | Cabbage casserole with “pulled oats” (oats and bean protein product), 345 g  Lingonberries, 30 g  Oat bread, margarine, tomato, 175 g | “Beanit” Bolognese (fava bean protein product), oat pasta, 375 g  Tomato, 125 g  Cottage cheese, 80 g | Spinach broccoli soup, 500 g  Rye Bread, margarine, red pepper, cucumber, 150 g |
| Snack | Viili (Finnish fermented milk product), apple, bilberries and nuts, 385 g  Rye bread, margarine, cucumber, red pepper 75 g | Lingonberry smoothie, 255 g  Rye bread, margarine cucumber, red pepper, 75g | Overnight oats, 230 g  Rye bread, cucumber, red pepper, margarine, 78 g |
| Dinner | Salmon chickpea patties, 150 g  Vegetable spelt, 185 g  Yogurt sauce, 60 g  Green salad with cucumber, 105 g | Vegetable patties, 175 g  Vegetables and barley, 185 g  Yogurt sauce, 60 g  Green salad, cucumber tomato, 125 g | Oven baked salmon, 135 g  Potato, 180 g  Yogurt sauce, 40 g  Broccoli, 150 g  Green salad with rapeseed oil,135 g |
| Evening snack | Overnight oats, 200 g  Oat bread, margarine, cucumber, tomato, 120 g  Cashew nuts, 10 g | Oat bread, margarine, cucumber, tomato, 90 g  Berry-banana-smoothie, 325 g  ½ Banana, 70 g | Oat bread, pesto, tomato, 135 g  Apple, 120 g |

Supplemental Table 2b. The goal and the actual values of macronutrients and salt in crossover meals of Healthy Nordic diet per day

|  | Goal | Day 1 | Day 2 | Day 3 |
| --- | --- | --- | --- | --- |
| Price, € | 7 | 8.64 | 7.58 | 8.11 |
| Energy, kcal | 2200 | 2209 | 2225 | 2173 |
| Carbohydrates, E% | 50 | 50.8 | 54.1 | 48 |
| Protein, E% | 20 | 18.4 | 17.9 | 20.7 |
| Fat, E% | 30 | 30.1 | 27.4 | 30.5 |
| Salt, grams | ~5 | 6 | 6.7 | 4.1 |
| Fiber, grams | >40 | 46.8 | 48 | 42.7 |
